# Incidence of intraoperative hypotension during non-cardiac surgery in community anesthesia practice: a retrospective observational analysis

**DOI:** 10.1186/s13741-023-00318-y

**Published:** 2023-06-24

**Authors:** Wael Saasouh, Anna L. Christensen, Fei Xing, Desirée Chappell, Josh Lumbley, Brian Woods, Monty Mythen, Richard P. Dutton

**Affiliations:** 1 Department of Anesthesiology, Detroit Medical Center, Detroit, MI USA; 2 NorthStar Anesthesia, Irving, TX USA; 3 Outcomes Research Consortium, The Cleveland Clinic, Cleveland, OH USA; 4 Mathematica, Washington, DC, USA; 5 University College London, London, UK; 6 US Anesthesia Partners, Dallas, TX USA; 7 Texas A&M College of Medicine, Bryant, TX USA

**Keywords:** Blood pressure, Hypotension, Hemodynamics, Quality improvement, Community anesthesia

## Abstract

**Background:**

Intraoperative hypotension (IOH) is well-described in the academic setting but not in community practice. IOH is associated with risk of postoperative morbidity and mortality. This is the first report of IOH in the community setting using the IOH measure definition from the Centers for Medicare and Medicaid Services Merit-based Incentive Payment System program. Objectives: To describe the incidence of IOH in the community setting; assess variation in IOH by patient-, procedure-, and facility-level characteristics; and describe variation in risk-adjusted IOH across clinicians.

**Methods:**

Design

Cross-sectional descriptive analysis of retrospective data from anesthesia records in 2020 and 2021.

Setting

Forty-five facilities affiliated with two large anesthesia providers in the USA.

Participants

Patients aged 18 years or older having non-emergent, non-cardiac surgery under general, neuraxial, or regional anesthesia. Cases were excluded based on criteria for the IOH measure: baseline mean arterial pressure (MAP) below 65 mmHg prior to anesthesia induction; American Society of Anesthesiologists (ASA) physical status classification of I, V, or VI; monitored anesthesia care only; deliberate induced hypotension; obstetric non-operative procedures; liver or lung transplant; cataract surgery; non-invasive gastrointestinal cases.

Main outcomes

IOH, using four definitions. Primary definition: binary assessment of whether the case had MAP < 65 mmHg for 15 min or more. Secondary definitions: total number of minutes of MAP < 65 mmHg, total area under MAP of 65 mmHg, time-weighted average MAP < 65 mmHg.

**Results:**

Among 127,095 non-emergent, non-cardiac cases in community anesthesia settings, 29% had MAP < 65 mmHg for at least 15 min cumulatively, with an overall mean of 12.4 min < 65 mmHg. IOH was slightly more common in patients who were younger, female, and ASA II (versus III or IV); in procedures that were longer and had higher anesthesia base units; and in ambulatory surgery centers. Incidence of IOH varied widely across individual clinicians in both unadjusted and risk-adjusted analyses.

**Conclusion:**

Intraoperative hypotension is common in community anesthesia practice, including among patients and settings typically considered “low risk.” Variation in incidence across clinicians remains after risk-adjustment, suggesting that IOH is a modifiable risk worth pursuing in quality improvement initiatives.

**Supplementary Information:**

The online version contains supplementary material available at 10.1186/s13741-023-00318-y.

## Background

Intraoperative hypotension (IOH) is associated with adverse myocardial, renal, and neurological outcomes; increased hospital length of stay; and postoperative mortality (Ahuja et al. 2020; An et al. 2019; Bijker and Gelb 2013; Bijker et al. 2012, 2009; Brady et al. 2020; Futier et al. 2017; Gregory et al. 2021; Gu et al. 2018; Huang et al. 2020; Maheshwari et al. 2020; Mathis et al. 2020; Monk et al. 2016; Radinovic et al. 2019; Salmasi et al. 2017; Sessler et al. 2019; Wesselink et al. 2018; Wijnberge et al. 2021). Associations are well-documented and consistent across observational studies and across multiple blood pressure thresholds (Ahuja et al. 2020; An et al. 2019; Sessler et al. 2019). However, they are generally studied at major academic centers, with limited data on IOH in community anesthesia practice where a significant portion of annual anesthetics are estimated to occur (Almanac 2021).

Anesthesia clinicians routinely monitor intraoperative blood pressure and maintain hemodynamic stability. The American Society of Anesthesiologists (ASA) standard for intraoperative blood pressure monitoring includes a minimum of one reading every 5 min (American Society of Anesthesiologists. Standards for Basic Anesthetic Monitoring 2010), and the Perioperative Quality Initiative consensus statement recommends maintaining systolic arterial pressure above 100 mmHg and mean arterial pressure (MAP) above 60–70 mmHg (Sessler et al. 2019). To aid in monitoring and reducing IOH, a risk-adjusted, clinician-level measure was developed to assess intraoperative hemodynamic control during non-emergent non-cardiac surgery (Christensen et al. 2021). This measure—the “IOH measure”—assesses the percent of cases with MAP below 65 mmHg for 15 min or more cumulatively, risk-adjusted for patient age, sex, body-mass-index (BMI), ASA physical status, and surgery length. The IOH measure has been reported in the Centers for Medicare and Medicaid Services (CMS) Merit-based Incentive Payment System (MIPS) as a Qualified Clinical Data Registry measure since 2020. While there are many ways to define IOH (Bijker et al. 2007), the IOH measure is one validated method using blood pressure readings recorded in the anesthesia information management system (AIMS). It is also supported by the ASA and utilized as a federal quality measure.

The aims of this manuscript are to assess the incidence of IOH in community anesthesia practice, to describe variations in the incidence of IOH across subgroups of patients and surgical procedures, and to assess whether incidence of IOH varies among clinicians after controlling for risk factors.

## Methods

### Study approval

The Health Media Lab Institutional Review Board (IRB) reviewed this study and determined it met criteria for IRB exemption, in accordance with the US Code of Federal Regulations for the Protection of Human Subjects, 45 CFR 46.104. The requirement for written informed consent was waived by the IRB. The study was conducted in accordance with the appropriate Enhancing the QUAlity and Transparency Of health Research (EQUATOR) guidelines.

### Data sources

Two anesthesia practices (NorthStar Anesthesia [NSA] and US Anesthesia Partners [USAP]) collaborated on this study. Each practice extracted retrospective data from their AIMS, electronic health records (EHR), and/or billing systems on non-emergent, non-cardiac cases from 2020 and 2021, from a subset of facilities that store the perioperative blood pressure readings in the AIMS. One practice used their qualified clinical data registry vendor to supply data, and the other relied on their quality improvement department. Researchers from outside the practices determined which covariates each practice could provide in structured fields. Data included: blood pressure readings taken approximately every 1–5 min, patient characteristics (age, sex, BMI, ASA physical status classification), procedure characteristics (anesthesia procedure code, surgery length), and facility type (ambulatory surgical center [ASC] or hospital). We did not have access to information on the anesthesia coverage model or anesthesia clinician type (e.g., anesthesiologist, certified registered nurse anesthetist), although the majority of cases were performed in an anesthesia care team model with collaboration between anesthesiologists and anesthetists. We also did not collect data regarding vasoactive medication use or choice of anesthetic agent. The data were reviewed and put into a uniform structure and thorough data quality checks were conducted.

### Study sample

Surgical cases were included in the analysis if they met criteria for the IOH measure: patients aged 18 years or older having non-emergency, non-cardiac surgeries (including elective and urgent surgeries) under general, neuraxial, or regional anesthesia care. Cases also must have included one of the 220 anesthesia CPT codes eligible for the IOH measure. Surgical cases were excluded from this analysis if they met exclusion criteria for the IOH measure: baseline MAP below 65 mmHg; ASA physical status classification I, V, or VI; monitored anesthesia care only; induced hypotension (using CPT add on code 99,135); obstetric non-operative procedures; liver or lung transplant; cataract surgery; non-invasive gastrointestinal cases.

### Outcomes

We assessed IOH using four definitions. The primary definition aligned with the IOH measure and is a binary assessment of whether the case had MAP < 65 mmHg for at least 15 min, cumulatively. Secondary definitions of IOH included: the total number of minutes of MAP < 65 mmHg, the total area under MAP of 65 mmHg, and the time-weighted average (TWA) MAP < 65 mmHg. Total area under 65 mmHg was calculated as follows: for each blood pressure episode below 65 mmHg, we calculated the area as the units of mmHg below 65 mmHg (depth of hypotension), multiplied by the length (in minutes) between the index blood pressure reading and the next reading (duration of hypotension). The areas were then summed across all hypotensive episodes during the case to arrive at the total area (Maheshwari et al. 2018). TWA MAP < 65 mmHg was calculated as the total area under 65 mmHg divided by the surgery length in minutes. Because blood pressure data can contain artifactual values, we dropped readings documented as an artifact by the clinician, systolic blood pressure (SBP) ≥ 300 mmHg or ≤ 20 mmHg, diastolic blood pressure (DBP) ≤ 5 mmHg or ≥ 225 mmHg, SBP, and DBP within 5 mmHg, MAP ≤ 30 mmHg or ≥ 250 mmHg.

To calculate IOH, each blood pressure reading was attributed to the period from the time the reading was recorded to the time of either the next reading or the anesthesia end time, capped at 5 min maximum.

### Statistical analysis

We assessed the incidence of IOH in the full sample using the four definitions described above. To describe the depth and duration of hypotension among cases that met the primary definition, we calculated the secondary definitions, stratified by whether cases met the primary definition. To determine the variation in hypotension across subgroups, we calculated IOH by patient characteristic, procedure characteristic, and facility type. ASA physical status classification was a proxy for patient comorbidities. Anesthesia base units, which are procedure-specific values that partially determine anesthesia payments, were a proxy for procedure complexity. Chi-square tests assessed the statistical significance of the variation. We also calculated IOH for the 10 most common anesthesia procedure codes in the sample.

For analyses of clinician-level variation in IOH, we attributed each case to the clinician(s) included on the anesthesia record. We limited to clinicians with at least 30 cases during the study period to ensure reliable estimates, based on data-driven recommendations from the measure developers (Christensen et al. 2021). For each clinician, we calculated the unadjusted and risk-adjusted versions of the IOH measure and the TWA. The risk-adjusted IOH measure is calculated as an observed to expected ratio (O:E ratio); its development and validation has been described previously (Christensen et al. 2021). It is equal to the count of “observed” or actual cases of IOH during the study period, divided by the number of cases of IOH that were “expected” during the study period based on their case mix. The expected number is determined by a logistic regression model with covariates for patient age, sex, BMI, ASA physical status, and surgery length. To calculate the risk-adjusted measure in a subset of cases with missing BMI, we imputed missing values as the median BMI for the patient’s age and sex combination.

To assess whether clinicians with different rates of hypotension had different case mixes, we categorized clinicians by their risk-adjusted rates of hypotension (O:E ratios of less than 0.8, 0.8 to 1.2, greater than 1.2), then described the patient, procedure, and facility characteristics of the cases in each clinician group.

Data management and statistical analyses were performed using R software. All statistical tests were two-tailed, and *P* values less than 0.01 were considered significant.

## Results

From NSA and USAP, respectively, the sample included 127,095 surgical cases (43% and 57%) from 45 facilities (10 and 35) in the midwest, southeast, and southwest regions of the USA. Cases were conducted by 663 anesthesia clinicians (49% and 51%). Supplemental Figure S1 shows the sample selection flowchart. Sample characteristics are shown in Table 1. Nearly half of cases were among patients aged 40 to 64 years. Forty-seven percent of patients were ASA physical status II; 46% were ASA physical status III. The most common surgical duration was 60–199 min (42%). Eighty-seven percent of surgeries occurred in a hospital setting, and 13% occurred in an ASC.

**Table 1:** Sample characteristics (*n* = 127,095)

	*n* (%)
Total	
Age group	
18–39 years	23,702 (18.6)
40–64 years	61,848 (48.7)
≥ 65 years	41,545 (32.7)
Sex	
Female	69,964 (55.1)
Male	57,105 (44.9)
ASA^a^ physical status	
II	59,753 (47.0)
III	58,051 (45.7)
IV	9291 (7.3)
Surgery length	
< 60 min	24,068 (18.9)
60–119 min	53,484 (42.1)
120–179 min	27,338 (21.5)
180–239 min	11,724 (9.2)
240–299 min	5272 (4.1)
300 + min	5209 (4.1)
Anesthesia type	
General	122,802 (96.6)
Regional	3617 (2.9)
Neuraxial	676 (0.5)
Anesthesia complexity	
Base units 0 to 5	66,169 (52.1)
Base units 6 to 10	49,153 (38.7)
Base units 11 or greater	10,854 (8.5)
Facility type	
Ambulatory surgical center	16,750 (13.2)
Hospital	110,345 (86.8)

26 patients had missing data for sex; 919 patients had missing data for anesthesia complexity

^a^*ASA* American Society of Anesthesiologists

The incidence of IOH per the primary outcome (MAP < 65 mmHg for at least 15 min, cumulatively) was 29.0% (Table 2). Cases had a mean of 12.4 min of MAP < 65 mmHg. The mean area under MAP of 65 mmHg was 65.4 mmHg*min, and the mean time weighted average below a MAP of 65 mmHg was 0.67 mmHg. Cases that met the IOH measure numerator had an average of 36.2 min < 65 mmHg.

**Table 2:** Incidence of intraoperative hypotension (unadjusted), *n* = 127,095 cases

	MAP^*a*^ < 65 for 15 + min	Total time MAP < 65 (mean)	Area under MAP of 65 (mean)	Time weighted average MAP < 65 (mean)
All cases (*n* = 127,095)	29.0%	12.4 min	65.4 mmHg*min	0.67 mmHg
Cases stratified by Intraoperative Hypotension measure numerator status (MAP < 65 for 15 + min)				
Cases that meet numerator (*n* = 36,838)	100%	36.2 min	194.3 mmHg*min	1.83 mmHg
Cases that do not meet numerator (*n* = 90,257)	0%	2.7 min	12.8 mmHg*min	0.19 mmHg

^a^*MAP* mean arterial pressure

Incidence of IOH (defined as MAP < 65 mmHg for at least 15 cumulative minutes) differed by patient age, sex, ASA physical status, surgery length, anesthetic type, anesthesia base units, and facility type (Table 3; all Chi-square tests < 0.01). Across all four definitions of IOH, younger patients (age 18 to 39) had more IOH than older patients (age 40 to 64 and ≥ 65), and females experienced more IOH than males. Patients with ASA physical status II had the highest incidence of MAP < 65 mmHg for 15 min and had the most minutes of MAP < 65 mmHg; however, patients with ASA physical status IV had the highest area under MAP of 65 mmHg and highest time weighted average MAP below 65 mmHg. Using three definitions of IOH, the incidence increased steadily with increasing surgery length; however, once time-weighted, patients with surgeries between 60 and 119 min long had the greatest time-weighted average MAP < 65 mmHg. Cases with regional anesthesia had more IOH than those with general or neuraxial anesthesia for three of the definitions of IOH. Using three definitions of IOH, the incidence increased with increasing anesthesia complexity (base units); however, once time-weighted, cases with the lowest complexity (base units 0 to 5) had the greatest time-weighted average MAP < 65 mmHg.

**Table 3:** Incidence of intraoperative hypotension (unadjusted), by patient characteristics, surgery length, and facility type (*n* = 127,095)

	MAP^*a*^ < 65 for 15 + min (%)	Total time MAP < 65 (mean min)	Area under MAP of 65 (mean mmHg*min)	Time weighted average MAP < 65 (mean mmHg)
Total	29.0%	12.4	65.4	0.67
Age group*				
18–39 years	32.1%	15.3	79.2	0.82
40–64 years	28.4%	12.0	62.2	0.62
≥ 65 years	28.0%	11.5	62.3	0.65
Sex*				
Female	32.3%	14.0	74.6	0.75
Male	24.9%	10.5	54.1	0.56
ASA^*b*^ physical status*				
II	31.0%	13.5	65.6	0.67
III	27.2%	11.4	63.8	0.64
IV	27.2%	11.8	73.7	0.79
Surgery length*				
< 60 min	12.5%	4.4	23.3	0.65
60–119 min	27.4%	10.1	53.1	0.74
120–179 min	35.7%	15.3	80.0	0.63
180–239 min	40.5%	19.5	104.3	0.57
240–299 min	42.0%	23.1	122.9	0.52
300 + min	46.4%	31.3	163.2	0.51
Anesthesia type*				
General	28.6%	12.3	65.2	0.67
Regional	43.2%	18.7	80.2	0.64
Neuraxial	15.2%	5.7	25.1	0.32
Anesthesia complexity*				
Base units 0 to 5	28.2%	12.2	61.0	0.75
Base units 6 to 10	29.1%	12.2	66.1	0.58
Base units 11 or greater	33.5%	15.2	89.8	0.53
Facility type*				
Ambulatory surgical center	30.9%	13.6	60.1	0.69
Hospital	28.7%	12.2	66.2	0.66

26 patients had missing data for sex; 919 patients had missing data for anesthesia complexity

^*^Chi-squared test *p* < 0.01 for primary outcome (MAP < 65 mmHg for 15 + min)

^a^*MAP* mean arterial pressure

^b^*ASA* American Society of Anesthesiologists

IOH was slightly more common in ASCs than hospitals, across three of the four definitions shown in Table 3.

Among the 10 most common anesthesia procedures, incidence of IOH (defined as MAP < 65 mmHg for at least 15 min) varied from 16.9% during anesthesia for hand cases to 36.5% during anesthesia for extensive spine procedures (Table 4). The TWA ranged from 0.44 mmHg in anesthesia for lower abdominal cases including laparoscopy, to 0.96 mmHg in anesthesia for procedures on the skin (Table 4).

**Table 4:** Incidence of intraoperative hypotension for the top 10 anesthesia procedures in sample

CPT^a^	Anesthesia base units for CPT (in 2022)	n cases	MAP^b^ < 65 for 15 + min (%)	Total time MAP < 65 (mean min)	Area under MAP of 65 (mean mmHg* min)	Time weighed average MAP < 65 (mean mmHg)
00840: Anesthesia for intraperitoneal procedures in lower abdomen including laparoscopy; NOS^*c*^	6	10,532	25.2	10.2	52.0	0.44
00790: Anesthesia for intraperitoneal procedures in upper abdomen including laparoscopy; NOS	7	9931	23.0	9.2	49.2	0.45
00400: Anesthesia for procedures on the integumentary system on the extremities, anterior trunk and perineum; NOS	3	7723	29.8	11.6	62.4	0.96
00670: Anesthesia for extensive spine and spinal cord procedures (e.g., spinal instrumentation or vascular procedures)	13	7013	36.5	15.9	95.6	0.54
01480: Anesthesia for open procedures on bones of lower leg, ankle, and foot; NOS	3	6304	30.2	12.2	55.8	0.67
00300: Anesthesia for all procedures on the integumentary system, muscles and nerves of head, neck, and posterior trunk, NOS	5	4146	35.6	16.5	88.7	0.90
01402: Anesthesia for open or surgical arthroscopic procedures on knee joint; total knee arthroplasty	7	4144	34.5	14.8	65.3	0.51
01400: Anesthesia for open or surgical arthroscopic procedures on knee joint; NOS	4	3925	17.4	7.5	35.3	0.47
01630: Anesthesia for open or surgical arthroscopic procedures on humeral head and neck, sternoclavicular joint, acromioclavicular joint, and shoulder joint; NOS	5	3706	27.8	11.2	52.8	0.56
01810: Anesthesia for all procedures on nerves, muscles, tendons, fascia, and bursae of forearm, wrist, and hand	3	3570	16.9	6.9	32.0	0.57

^a^*CPT* Current Procedural Terminology

^b^*MAP* mean arterial pressure

^c^ *NOS* not otherwise specified

### Clinician-level analyses

**Fig. 1:**
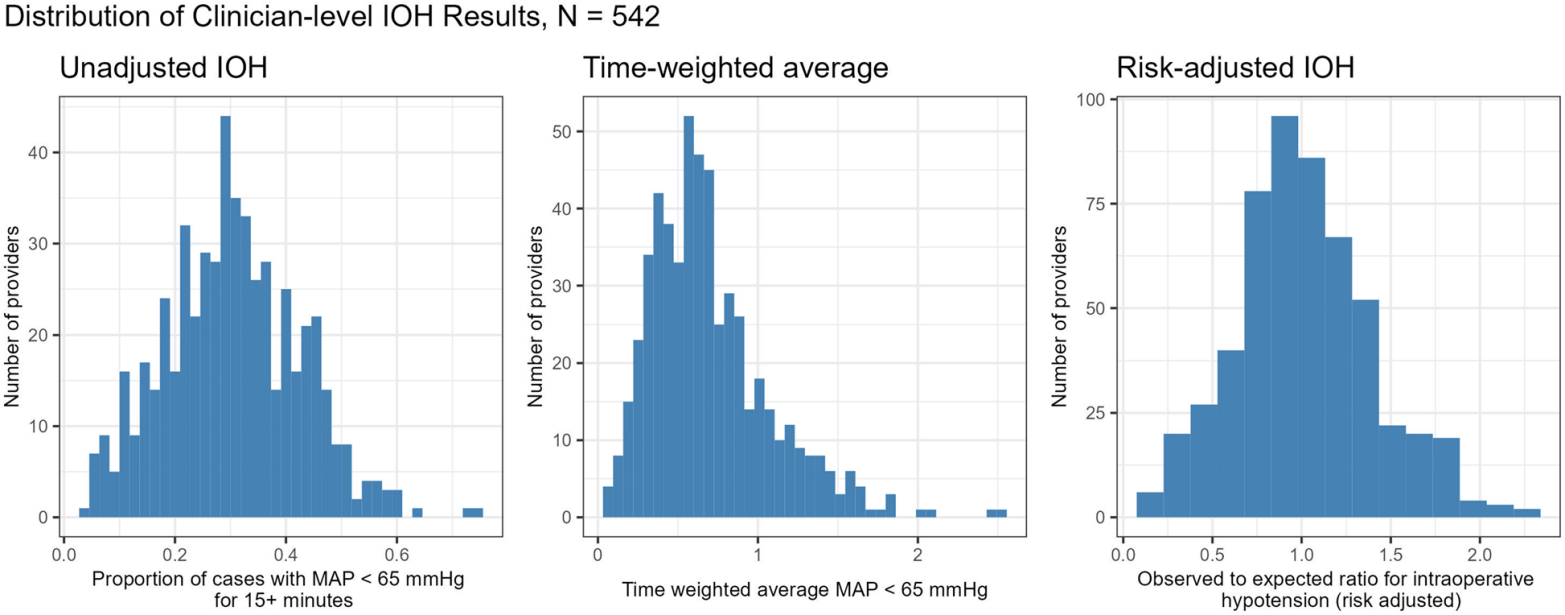
Distribution of anesthesia clinicians’ incidence of intraoperative hypotension, measured 3 ways. IOH: intraoperative hypotension; MAP: mean arterial pressure; TWA: time-weighted average

The incidence of IOH varied widely across clinicians for each of the 3 definitions of the outcome we assessed: percent of cases with MAP < 65 mmHg for ≥ 15 min (unadjusted); TWA; and risk-adjusted intraoperative hypotension using the IOH measure (Fig. 1). Clinicians varied from having 3.4 to 74.3% of their cases having MAP < 65 mmHg for at least 15 min, with a median incidence of 29.9% (not shown).

Clinicians who had at least 20% more cases of IOH than expected (O:E ≥ 1.2) had cases that were slightly younger, slightly more female, and slightly lower BMI and ASA physical status than clinicians with O:E ratios less than 0.8. Median surgery length was 2 min longer for clinicians with more hypotension than expected, compared to those with less hypotension than expected. Clinicians with more IOH had a greater proportion of cases in ASCs (19% in ASCs for clinicians with O:E ratios > 1.2; 8% in ASCs for those with O:E ratios 0.8–1.2; 7% in ASCs for those with O:E ratios < 0.8) (Supplemental material Tables S1–S6).

## Discussion

In our sample of community anesthesia practice, there was a substantial occurrence of IOH using the previously-defined IOH measure, with 29% of cases having MAP < 65 mmHg for 15 min or longer. IOH has been reported in 5–99% of surgical cases (Bijker and Gelb 2013; Warner and Monk 2007; Weinberg et al. 2022). Limiting to studies that use a similar MAP threshold of 65 mmHg, the incidence of IOH ranges from 19 to 88% for MAP < 65 mmHg for at least 1 min (Bijker and Gelb 2013; Gregory et al. 2021; Shah et al. 2020), 45–49% for at least 5 min (Bijker and Gelb 2013; Shah et al. 2020), and 31% for at least 10 min (Bijker and Gelb 2013; Shah et al. 2020). Most previous studies are from major academic institutions. The incidence we observed was surprising; we presumed our study population in community anesthesia was lower risk compared to previous studies that focused on academic hospitals.

Counter-intuitively, patients under age 40 experienced more IOH than those over 65. This aligns with some (Shah et al. 2020) but not all (Gregory et al. 2021; Dai et al. 2020; Wickham et al. 2022) published reports. Clinicians may be less likely to intervene on younger patients perceived to tolerate lower pressures (Franck et al. 2011). While older patients are more predisposed to hemodynamic disturbances, it is unclear whether this is predictive of adverse outcomes (Gregory et al. 2021; Bonnet et al. 2020). IOH occurred more commonly in females than males, which aligns with published data (Gregory et al. 2021; Kalezic et al. 2013; Wickham et al. 2022). We did not report variation by BMI due to missing data. Patients with ASA physical status II experienced more minutes of IOH and were more likely to have at least 15 min of MAP < 65 mmHg, compared to patients with ASA status III or IV. This aligns with some published reports (Kalezic et al. 2013) but contrasts with others (Wickham et al. 2022). This pattern may reflect sicker patients being managed by more experienced clinicians and in better-equipped settings. Practically, sicker patients are more likely to be monitored more frequently—or more invasively—and hypotensive episodes may be shorter-lived because of earlier or more aggressive intervention (Bijker et al. 2009). However, ASA IV patients had the highest area under MAP < 65 mmHg and highest time weighted average MAP < 65 mmHg, suggesting that this group had more severe drops in blood pressure when they occurred.

Incidence of IOH increased with surgical duration across most definitions, a finding in agreement with some (Bijker et al. 2009; Südfeld et al. 2017; Sun et al. 2015) but not all (Ahuja et al. 2020; Franck et al. 2011; Kalezic et al. 2013) reports. The time-weighted average of IOH was higher among shorter surgeries and highest among surgeries lasting 1–2 h. This may be because anesthetic induction is a period of risk for IOH, and the induction period makes up a larger proportion of shorter surgeries versus longer surgeries.

The majority (96.6%) of analyzed cases qualified as general anesthetics with 28.6% incidence of IOH. Reliable comparisons between different anesthetics cannot be made since anesthetic type was determined by the highest level anesthetic administered. Anesthesia base units were used as a practical surrogate of anesthetic complexity. Most cases were lower complexity (0–5 base units) or intermediate complexity (6–10 base units). High complexity cases (11 or greater base units) were least common but had the highest incidence of MAP < 65 for at least 15 min (33.5%) compared to intermediate (29.1%) and low complexity (28.2%) cases. However, in the time-weighted definition of IOH, the lowest complexity cases (base units 0 to 5) had the highest TWA MAP < 65 mmHg.

Incidence of IOH was higher in ASCs than in hospitals, using three of the four definitions of IOH, including TWA. Our median surgery length was only 21 min shorter in ASCs than in hospitals (65 versus 86 min) (Supplemental material Table S7). This result may reflect trends in management of IOH in ASCs, where patients are presumed to be healthier (less intensively monitored), or sites are less prepared to escalate IOH treatment.

The incidence of IOH varied across clinicians. Counter-intuitively, clinicians with worse scores on the risk-adjusted IOH measure were generally caring for younger patients with lower ASA physical status and were more likely in an ASC. This suggests that variation in IOH at least partially results from differences in clinician behavior or assessment of risk, as opposed to simply being driven by patient risk factors. Hence, IOH—generally attributed to pre-existing conditions and higher-risk procedures (Mathis et al. 2020; Wickham et al. 2022)—may be an appropriate target for quality improvement.

This analysis has limitations. The data represent a convenience sample from two large anesthesia practices. Despite including 45 facilities in multiple U.S. regions, data may not be nationally representative. Our study population is limited to non-emergent non-cardiac surgical procedures and the facilities that contributed data do not use unified hemodynamic monitoring protocols. Considering the inherent limitations of retrospective and descriptive analyses, it is difficult to determine differences in IOH treatment among clinicians. We were unable to adjust the analysis based on combination anesthetics (i.e., general + regional) since the highest anesthetic intervention was allocated to each case.

BP values were either manually entered or automatically captured in the electronic record. It is reasonable to assume that manually recorded vitals would be artificially “smoothened,” but they may also omit obvious artifacts that an electronic system would include (Franck et al. 2011). However, there was not a clinically meaningful difference in IOH incidence across modes of data entry.

The IOH measure used for the clinician-level analysis is only risk-adjusted for five factors. To minimize reporting burden and limit chart reviews, the current model was limited to variables available to the anesthesia registries that calculate the measure: age, gender, BMI, ASA physical status, and surgical duration. Also, while some clinicians recommend using relative drops in blood pressure from a predefined, patient-specific baseline as a means to individualize hemodynamic management (Futier et al. 2017); absolute thresholds have been shown to be similarly predictive of morbidity (Mathis et al. 2020; Salmasi et al. 2017; Mascha et al. 2015). It is plausible that MAP < 65 mmHg is equivalent to a ≥ 20% deviation from baseline for most patients. Based on these different definitions and thresholds, it is difficult to determine which of our four IOH definitions is the best target for intervention.

We did not have outcome data to study acknowledging that IOH is consistently associated with adverse outcomes. Based on published observational analyses, it is likely that the lower the blood pressure, and the longer the episode of hypotension, the greater the risk for adverse outcomes (An et al. 2019; Bijker et al. 2009; Brady et al. 2020; Futier et al. 2017; Gregory et al. 2021; Radinovic et al. 2019; Salmasi et al. 2017; Sessler et al. 2019; Wesselink et al. 2018; Wijnberge et al. 2021; Maheshwari et al. 2018; Wickham et al. 2022). We also did not have access to comorbidity or hospital details for our population, which would have made our results more robust. Community practice sites included in this analysis have limited resources for research, if any, and requesting that granular data was deemed not feasible within the scope of this project.

Despite limitations, this study contributes by describing IOH rates in a community anesthesia setting and suggests that the burden of IOH is not limited to complex cases in academic hospitals. That IOH is more common in “lower risk” patients/settings and that it varies across clinicians, even after risk adjustment, suggests that it is modifiable (not solely determined by the patient and case risk) and a potential target for quality improvement. By instituting educational and systematic quality efforts, IOH could be amenable to mitigation, potentially improving outcomes for many patients. Policy changes may also provide incentives to reduce IOH. A recent government report on adverse events in hospitals found that hypotension was the most common harm event related to surgeries and procedures. The report recommended that CMS update its lists of hospital acquired conditions (HAC) for the HAC Reduction Program and the Deficit Reduction Act HAC list to capture common, preventable, and high-cost harm events, potentially including IOH (Grimm 2018).

## Conclusions

Intraoperative hypotension is common in community anesthesia practice, including among patients and settings typically considered “low risk.” Variation in incidence across clinicians remains after risk-adjustment, suggesting that IOH is a modifiable risk worth pursuing in quality improvement initiatives.

## Supplementary Information

**Additional file 1: Figure S1.** Sample selection. **Table S1.** Patient age distributionby clinician score group. **Table S2.** Percent female by clinician score group. **Table S3.** Mean patient BMI by clinician score group. **Table S4.** Mean ASA physical status by clinician score group. **Table S5.** Surgery length distributionby clinician score group. **Table S6.** Facility type by clinician score group. **Table S7.** Surgery length distributionby facility type.

## Data Availability

The data that support the findings of this study are available from NorthStar Anesthesia and US Anesthesia Partners. Restrictions apply to the availability of these data, which were used under license for the current study, and so are not publicly available. Data are however available from the authors upon reasonable request and with permission of NorthStar Anesthesia and US Anesthesia Partners.

## Abbreviations

AIMS: Anesthesia information management system
ASA: American Society of Anesthesiologists
ASA: American Society of Anesthesiologists
ASC: Ambulatory surgical center
BMI: Body-mass-index
CMS: Centers for Medicare and Medicaid Services
DBP: Diastolic blood pressure
HAC: Hospital acquired conditions
HER: Electronic health records
IOH: Intraoperative hypotension
IRB: Institutional Review Board
MAP: Mean arterial pressure
MAP: Mean arterial pressure
MIPS: Merit based Incentive Payment System
NSA: NorthStar Anesthesia
O:E ratio: Observed to expected ratio
SBP: Systolic blood pressure
TWA: Time-weighted average
USAP: US Anesthesia Partners
