# Supplementary material for: Incidence of intraoperative hypotension during non-cardiac surgery in community anesthesia practice: a retrospective observational analysis
Source: Perioper Med (Lond). 2023 Jun 24;12:29. doi: 10.1186/s13741-023-00318-y (PMC10290388; doi:10.1186/s13741-023-00318-y)
Supplement: Supplementary file 1 — Additional file 1: Figure S1. Sample selection. Table S1. Patient age distributionby clinician score group. Table S2. Percent female by clinician score group. Table S3. Mean patient BMI by clinician score group. Table S4. Mean ASA physical status by clinician score group. Table S5. Surgery length distributionby clinician score group. Table S6. Facility type by clinician score group. Table S7. Surgery length distributionby facility type. [file 13741_2023_318_MOESM1_ESM.docx]

**Supplement**

**Figure S.1. Sample selection**

Cases with necessary case-level data and blood pressure readings

(n= 236,599)

Excluded from IOH measure for one or more reason below

(n= 14,090)

-Baseline MAP < 65 mmHg (n= 2,250)

-ASA 1, 5, 6 (n = 12,229)

-Induced hypotension (n= 0)

[Note: numbers will not sum to total because some patients had more than one exclusion]

Excluded because missing ASA physical status

(n= 15,395)

Cases that meet the inclusion criteria

-Age>=18

-Non-emergent surgery

-General, regional, or neuraxial anesthesia

-Relevant anesthesia CPT code

(n= 156,580)

Case included in analysis

(n= 127,095)

**Table S.1. Patient age distribution (years) by clinician score group**

| **Clinician group** | **25th percentile** | **50th percentile** | **75th percentile** | **Mean*** |
| --- | --- | --- | --- | --- |
| O:E ratio <0.8 | 44 | 58 | 69 | 56.3 |
| O:E ratio 0.8 to 1.2 | 44 | 57 | 67 | 55.6 |
| O:E ratio >1.2 | 43 | 56 | 67 | 54.8 |

Note: O:E ratio= observed to expected ratio

*1-way ANOVA p < 0.01

**Table S.2. Percent female by clinician score group**

| **Clinician group** | **% Female*** |
| --- | --- |
| O:E ratio <0.8 | 55.4 |
| O:E ratio 0.8 to 1.2 | 54.7 |
| O:E ratio >1.2 | 57.5 |

Note: O:E ratio= observed to expected ratio

*Chi-squared p < 0.01

**Table S.3. Mean patient BMI by clinician score group**

| **Clinician group** | **Mean BMI*** |
| --- | --- |
| O:E ratio <0.8 | 29.3 |
| O:E ratio 0.8 to 1.2 | 28.5 |
| O:E ratio >1.2 | 28.5 |

Note: O:E ratio= observed to expected ratio

*1-way ANOVA p < 0.01

**Table S.4. Mean ASA physical status by clinician score group**

| **Clinician group** | **Mean ASA physical status*** |
| --- | --- |
| O:E ratio <0.8 | 2.674 |
| O:E ratio 0.8 to 1.2 | 2.657 |
| O:E ratio >1.2 | 2.541 |

Note: O:E ratio= observed to expected ratio

*1-way ANOVA p < 0.01

**Table S.5. Surgery length distribution (minutes) by clinician score group**

| **Clinician group** | **25th percentile** | **50th percentile** | **75th percentile** | **Mean*** |
| --- | --- | --- | --- | --- |
| O:E ratio <0.8 | 50 | 83 | 129 | 101.2 |
| O:E ratio 0.8 to 1.2 | 54 | 90 | 147 | 113.1 |
| O:E ratio >1.2 | 50 | 85 | 140 | 107.6 |

Note: O:E ratio= observed to expected ratio

*1-way ANOVA p < 0.01

**Table S.6. Facility type by clinician score group**

| **Clinician group** | **% of cases in ASCs*** | **% in hospitals** |
| --- | --- | --- |
| O:E ratio <0.8 | 7% | 93% |
| O:E ratio 0.8 to 1.2 | 8% | 92% |
| O:E ratio >1.2 | 19% | 81% |

Note: ASC = ambulatory surgical center; O:E ratio= observed to expected ratio

*Chi-squared p < 0.01

**Table S.7. Surgery length distribution (minutes) by facility type**

| **Facility type** | **10th percentile** | **25th percentile** | **50th percentile**  **(i.e median)** | **75th percentile** | **90th percentile** | **Mean (SD)*** |
| --- | --- | --- | --- | --- | --- | --- |
| ASC | 30 | 40 | 65 | 100 | 150 | 82 (63) |
| Hospital | 33 | 53 | 86 | 135 | 202 | 105 (77) |

Note: ASC = ambulatory surgical center; O:E ratio= observed to expected ratio

*Chi-squared p < 0.01
